# Lack of association of genetic variation in chromosome region 15q14-22.1 with type 2 diabetes in a Japanese population

**DOI:** 10.1186/1471-2350-9-22

**Published:** 2008-03-27

**Authors:** Yuka Yamaguchi, Maki Moritani, Toshihito Tanahashi, Dai Osabe, Kyoko Nomura, Yuka Fujita, Parvaneh Keshavarz, Kiyoshi Kunika, Naoto Nakamura, Toshikazu Yoshikawa, Eiichiro Ichiishi, Hiroshi Shiota, Natsuo Yasui, Hiroshi Inoue, Mitsuo Itakura

**Affiliations:** 1 Division of Genetic Information, Institute for Genome Research, The University of Tokushima, 3-18-15, Kuramoto-cho, Tokushima, 770-8503, Japan; 2 Department of Bioinformatics, Division of Life Science Systems, Fujitsu Limited, 1-5-2, Higashishinbashi, Minato-ku, Tokyo, 105-7123, Japan; 3 Department of Endocrinology and Metabolism, Kyoto Prefectural University of Medicine Graduate School of Medical Science, 465, Kajii-cho, Hirokoji-Kawaramachi, Kamigyo-ku, Kyoto, 602-8566, Japan; 4 New Industry Creation Hatchery Center, Tohoku University, Aramaki, Aoba-ku, Sendai, Miyagi, 980-8579, Japan; 5 Department of Ophthalmology and Visual Neuroscience, Institute of Health Bioscience, The University of Tokushima, 770-8503, Japan; 6 Department of Orthopedics, Institute of Health Biosciences, The University of Tokushima, 770-8503, Japan

## Abstract

**Background:**

Chromosome 15q14-22.1 has been linked to type 2 diabetes (T2D) and its related traits in Japanese and other populations. The presence of T2D disease susceptibility variant(s) was assessed in the 21.8 Mb region between *D15S118 *and *D15S117 *in a Japanese population using a region-wide case-control association test.

**Methods:**

A two-stage association test was performed using Japanese subjects: The discovery panel (Stage 1) used 372 cases and 360 controls, while an independent replication panel (Stage 2) used 532 cases and 530 controls. A total of 1,317 evenly-spaced, common SNP markers with minor allele frequencies > 0.10 were typed for each stage. Captured genetic variation was examined in HapMap JPT SNPs, and a haplotype-based association test was performed.

**Results:**

SNP2140 (rs2412747) (*C/T*) in intron 33 of the ubiquitin protein ligase E3 component n-recognin 1 (*UBR1*) gene was selected as a landmark SNP based on repeated significant associations in Stage 1 and Stage 2. However, the marginal *p *value (*p *= 0.0043 in the allelic test, OR = 1.26, 95% CI = 1.07–1.48 for combined samples) was weak in a single locus or haplotype-based association test. We failed to find any significant SNPs after correcting for multiple testing.

**Conclusion:**

The two-stage association test did not reveal a strong association between T2D and any common variants on chromosome 15q14-22.1 in 1,794 Japanese subjects. A further association test with a larger sample size and denser SNP markers is required to confirm these observations.

## Background

Insulin resistance in muscle, adipose, and liver tissues and impaired insulin secretion from pancreatic β cells contribute to the pathogenesis of type 2 diabetes (T2D) [1]. Analyses using a traditional candidate gene approach and, more recently, genome-wide association studies (GWAS) [2-7], have generated a large amount of data that have confirmed 11 genes with replicated association with T2D in Caucasians [8]. However, the assessment in Japanese T2D patients, who characteristically have a lower body mass index (BMI) and a lower fasting insulin level than Caucasians [9], has not been largely performed. These differences suggest that Japanese individuals with T2D might have a different genetic background from other populations, and susceptibility variant(s) or gene(s) for the development of T2D in Japanese can only be identified with genetic assessment. Because candidate regions supported by replicated linkage signals in Japanese and other populations are expected to map T2D susceptibility gene(s), a region-wide association test was used as an alternative affective approach with the availability of unbiased reliability, timeliness and cost efficiency.

Among a number of proposed candidate regions for T2D and its related traits [10], the chromosome 15q14-22.1 candidate region is supported by replicated linkage signals of non-overlapping samples from Japanese subjects. In three studies of genome-wide linkage scan of Japanese subjects [11-13], chromosome 15q14-22.1 showed significant evidence of linkage with T2D (LOD = 2.41) [12] and a maximum LOD score (MLS) of 3.91 for early onset T2D [11] (Table 1). This region overlaps candidate region found in other populations, including Mexican Americans [14] and Pima Indians [15], findings that were replicated in a subsequent study of Mexican Americans [16] (Table 1). This candidate region supported by replicated linkage signals is expected to contain susceptibility gene(s). In the absence of convincing comprehensive association data on chromosome 15q, the challenge remains to identify the disease susceptibility variant(s) or gene(s) that definitively contribute to T2D. We focused on chromosome 15q14-22.1 at 32.6–51.2 cM as the susceptibility region in Japanese.

**Table 1:** Summary of linkage scan on chromosome 15q14-22.1

**Population**	**Sample**	**Score**	**Phenotype**	**STS marker**	**Position (cM)**	**References (year)**
Japanese	224 ASP in 159 families, 359 individuals	1.57 MLS	T2D	*D15S994*	40.25	Mori Y et al. 2002 [11]
		3.91 MLS	T2D (Young-onset < 45)	*D15S994*	40.25	
		2.44 MLS	T2D (BMI < 30)	*D15S994*	40.25	
		1.50 MLS	T2D	*D15S118*	32.58	
		0.95 MLS	T2D	*D15S117*	51.21	
Japanese	256 ASP in 164 families, 368 individuals	2.41 LOD	T2D (BMI < 22)	*CYP19*	45.62	Iwasaki N et al. 2003 [12]
Japanese	102 ASP in 102 families	0.25 LOD	T2D	*D15S117*	51.21	Nawata H et al. 2004 [13]

Mexican Americans	330 ASP in 170 families, 408 individuals	1.50 MLS	T2D	*D15S119*	45.62	Hanis CL et al. 1996 [14]
Pima Indians	388 ASP in 109 families, 363 individuals	1.46 LOD	insulin secretion (IVGTT 10)	*D15S659*	43.47	Pratley RE et al. 1998 [15]
Mexican Americans	330 ASP in 170 families and 363 nondiabetic individuals	1.27 LOD	T2D	*D15S119*	45.62	Cox NJ et al. 1999 [16]
	interaction with D15S119 and CYP19	4.00 LOD	T2D	*D15S119+ CYP19*		

ASP, affected sib-pairs; MLS, maximum lod score; LOD, logarithm of odds; IVGTT 10, plasma insulin response at 10 min above the basal level during the intravenous glucose tolerance test; D15S119 + CYP19, D15S119 interaction with CYP19 (NIDDM1). Each report identified nominal evidence of linkage or significant evidence of linkage with LOD > 3.91. Location is shown only for loci with positive evidence of linkage on chromosome 15q. The position is based on the Marshfield recombination genetic map .

http://research.marshfieldclinic.org/genetics/Home/index.asp

In this study, a region-wide two-stage association test was used to perform a comprehensive examination of genetic variant(s), using 1,317 common SNPs, in a Japanese population comprised of 1,794 unrelated cases and controls.

## Methods

### Subjects

This study utilized samples obtained from a total of 1,794 Japanese subjects: 904 T2D cases and 890 controls. The majority of T2D patients was recruited from the outpatient clinic of Tokushima University Hospital, Kyoto Prefectural University Hospital and their affiliated hospitals. All T2D diagnoses were based on the 1985 World Health Organization criteria, and patients were clinically defined as having gradual adult onset of the disease with medication. Patients with clinical criteria for monogenic forms of diabetes were excluded. The clinical characteristics of these samples are presented in Table 2. The controls were recruited from healthy adult members of the general population, who were intensively checked for a negative family history of diabetes, normal HbA_1c _levels of < 5.8%, the absence of other diseases, and Japanese ancestry based on their birthplace information. The controls were primarily recruited from the Pharma SNP Consortium (nation-wide collection of Japanese control subjects) [17] and obtained through the Health Science Research Resources Bank of the Japanese Collection of Research/Japan Health Science Foundation. The subjects were placed in one of two independent panels: the discovery panel (Stage 1; 372 cases and 360 controls) and the replication panel (Stage 2; 532 cases and 530 controls).

**Table 2:** Characteristics of the 1,794 Japanese subjects used in the association tests

**Stage 1 samples (discovery panel)**	**Cases (n = 372)**	**Controls (n = 360)**
Gender (M/F)	188/184	148/212
Age	64.4 ± 10.3	46.3 ± 19.9
HbA_1C _(%)	7.39 ± 1.48	4.79 ± 0.36
BMI (kg/m^2^)	23.7 ± 3.5	22.0 ± 2.9
Age at onset < 50 (%)	175 (47.0)	-
≥50(%)	195 (52.5)	-
unknown (%)	2 (0.5)	-
Family history (%)	125 (33.6)	-
Diabetes in both parents	11	
Diabetes in one parent	110	
Diabetes in son or daughter	4	
Insulin therapy (+) (%)	112 (30.1)	-

**Stage 2 samples (replication panel)**	**Cases (n = 532)**	**Controls (n = 530)**

Gender (M/F)	246/286	284/246
Age	62.0 ± 10.7	37.8 ± 11.3
HbA_1C _(%)	7.47 ± 1.43	4.88 ± 0.33
BMI (kg/m^2^)	23.6 ± 3.3	22.4 ± 3.0
Age at onset < 50 (%)	244 (45.9)	-
≥50(%)	287 (53.9)	-
unknown (%)	1 (0.2)	-
Family history (%)	207 (38.9)	-
Diabetes in both parents	22	
Diabetes in one parent	180	
Diabetes in son or daughter	5	
Insulin therapy (+) (%)	172 (32.3)	-

**Stage 1 + Stage 2 combined samples**	**Cases (n = 904)**	**Controls (n = 890)**

Gender (M/F)	434/470	432/458
Age	63.0 ± 10.6	41.2 ± 15.9
HbA_1C _(%)	7.43 ± 1.45	4.84 ± 0.34
BMI (kg/m^2^)	23.6 ± 3.4	22.3 ± 3.0
Age at onset < 50 (%)	419 (46.4)	-
≥50(%)	482 (53.3)	-
unknown (%)	3 (0.3)	-
Family history (%)	332 (36.7)	-
Diabetes in both parents	33	
Diabetes in one parent	290	
Diabetes in son or daughter	9	
Insulin therapy (+) (%)	284 (31.4)	-

Genomic DNA was prepared from peripheral blood leukocytes or EB-virus-immortalized B lymphoblasts using a standard protocol. The study was approved by the Ethical Committee for Human Genome and Gene Research at the University of Tokushima in accordance with the tenets of the Declaration of Helsinki. Informed consent was obtained from all subjects prior to blood sampling, and all personal information and samples were anonymously analyzed for genotyping.

### Association testing of SNP markers and genotyping

At the onset of this study, there was no sufficient SNPs database, such as HapMap. Thus, we constructed a database of common Japanese SNPs based on the genotyping results of 45 unrelated Japanese control subjects (23 males and 22 females) in a whole genome in collaboration with Applied Biosystems (ABI) [18]. Out of 70,099 common (MAF > 0.15) gene-centric and intergenic SNPs made available by this project, we provisionally selected 1,450 evenly-spaced SNPs on chromosome 15q between two sequence-tagged site (STS) markers of *D15S118 *and *D15S117*.

The initial selection was based on the following criteria: 1) optimal suitability for the design of TaqMan high-throughput genotyping assays; 2) dense and evenly-spaced SNP coverage in both coding and intergenic regions; 3) definition of the gene location in a region between 10 kb upstream of the transcription start site and 10 kb downstream of the final exon; 4) a distance < 10 kb between adjacent SNPs; and 5) common SNPs with a MAF > 0.10. The criterion of a MAF > 0.10 was imposed subsequent to the selection of SNPs with a MAF > 0.15.

SNPs were assayed using TaqMan Universal PCR MasterMix (no UNG; ABI) as previously described [19-21]. The genotyping results were obtained in an auto-call mode, after which two researchers independently assessed the genotyping data. When the ratio of undetermined results to the total samples was greater than 2% (8/384 including four negative controls) or negative controls without DNA template were incorrectly detected, the genotyping was repeated. Genotyping results were compared to those obtained by direct sequencing as previously described [20] with perfect agreement between the two methods.

### Two-stage design of the association test

We performed a two-stage association test on 1,794 samples by randomly assigning them to two independent panels. In the first test, all of the SNPs were genotyped in Stage 1. Those exhibiting significant allelic or genotypic association (*p *< 0.05) were further examined in Stage 2. In each stage, the association was evaluated with two types of χ^2 ^test (allelic or genotypic) by 2 × 2 and 2 × 3 contingency tables for the status of cases or controls. Finally, the association was evaluated using the combined samples from both Stages.

The power of this case-control test was calculated based on MAF, a type 1 error rate (false-positive rate), and sample size with the PS program [22,23]. The standard Bonferroni's correction was used to evaluate the false-positive rate [24]. In addition, we used the false discovery rate (FDR) approach [25] as implemented with the FDR control program in the R language [26]. A logistic regression analysis to adjust for age, gender, and BMI was carried out using the SPSS program (ver.12, SPSS Japan Inc., Tokyo, Japan).

### Searching for novel SNPs and mutational screening of the *UBR1 *gene

The 47 coding exons, relevant intron-exon boundaries, and 5' and 3' UTR of *UBR1 *gene were screened for putative novel Japanese variants by direct sequencing the genomic DNA of 48 individuals (24 cases and 24 controls). All PCR products were treated with ExoSAP-IT (GE Healthcare, NJ, USA) and sequenced using BigDye Terminator Cycle Sequencing Kit, ver1.1 (ABI) in both directions on a 3730*xl *automated sequencer (ABI) according to the manufacturer's standard protocol.

### Estimation of linkage disequilibrium (LD) analysis for haplotype inference

Pairwise LD coefficients of |D'| and r^2 ^were assessed in Stage 1 samples with SNPAlyze ver 5.1 Pro software (DYNACOM, Japan) [27]. This analysis was performed under the assumption of Hardy-Weinberg equilibrium. Haplotype frequencies for multiple loci were estimated by the maximum-likelihood method with an expectation-maximization (EM) algorithm. Permutation *p *values were calculated by comparing haplotype frequencies between cases and controls on the basis of 10,000 replications. Haplotype-tagging SNPs (htSNPs) were determined with SNPAlyze ver 5.1 Pro.

### The amount of captured genetic variation based on HapMap JPT SNPs

HapMap JPT SNPs corresponding to 21.8 Mb were investigated [28]. Information on 29,728 SNPs within the target region was downloaded from the HapMap website (release 22) [29]. The density of the 1,317 SNPs (the finally selected SNPs from the 1,450 pre-HapMap SNPs) per 300-kb bin was compared against those in the HapMap JPT SNPs. In addition, we estimated the captured tag SNPs as proxies at r^2 ^> 0.7, 0.8, or 0.9 using Tagger program [30].

## Results

### Information of selected SNPs

One STS marker (*D15S118*) was located 3.9 Mb proximal to *D15S994*, which exhibited the highest evidence for linkage in the Japanese subjects with a LOD = 3.91. The second STS marker (*D15S117*) located 9.0 Mb distal to *D15S119*, was linked to T2D susceptibility in Mexican Americans with a LOD = 4.0 (Table 1). Therefore, a 21.8 Mb interval between *D15S118 *(32.6 cM, mp; 34.5 Mb) and *D15S117 *(51.2 cM, mp; 56.3 Mb) was chosen as the target region.

Out of 1,450 SNPs, quality control resulted in the exclusion of 39 SNPs that exhibited ambiguous genotyping qualities. After genotyping the remaining 1,411 SNPs, another 94 with a HWE *p *< 0.05 or a low MAF of < 0.10, an indication of insufficient power to detect statistical significance, were excluded. In the final selection of 1,317 SNPs, 974 were mapped within 152 genes (65.8% of the 231 genes based on the NCBI Build 36.1 [31] human genome assembly), and 343 were located in intergenic regions. The average MAF of the 1,317 SNPs was 0.32 ± 0.11 in Stage 1 samples, and the average spacing of the gene-centric SNPs was 9,169 bp per pair of SNPs across the 21.8 Mb.

### Captured genetic variation relative to HapMap JPT SNPs in the target region

**Figure 1:**
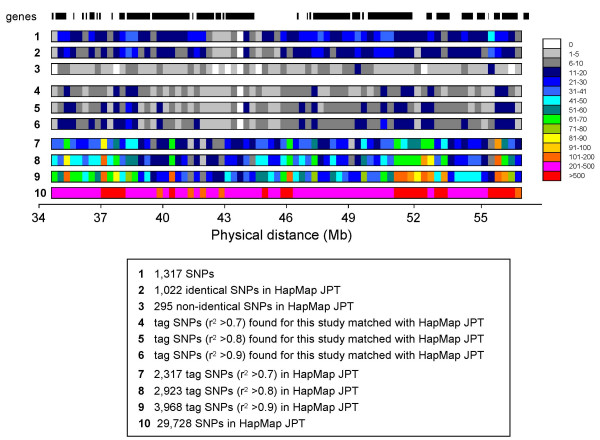
**SNP marker distribution in the target region**. Distribution of 1,317 SNPs selected for this study (1), 1,022 identical (2), and 295 non-identical (3) SNPs in the HapMap JPT database, and distribution of 29,728 SNPs in HapMap JPT (10) in every 300-kb bin against the physical distance in the target region. Number of tag SNPs (r^2 ^> 0.7, 0.8, and 0.9) found for this study matched with the r^2 ^> 0.7, 0.8, and 0.9 SNPs in HapMap JPT (4, 5, and 6) and number of tag SNPs (r^2 ^> 0.7, 0.8, and 0.9) among the HapMap JPT SNPs (7, 8, and 9). The vertical bars in the top panel show the position of RefSeq genes in this region.

Coverage of the 1,317 SNPs was more limited than that with 29,728 SNPs of HapMap JPT in the 21.8 Mb region: 1,022 SNPs (78%) of the 1,317 SNPs were present, while only 295 SNPs (22%) were not present in the 29,728 HapMap JPT (Figure 1), although they were registered in the public database [32] without information on allele frequency in the Japanese population. Figure 1 shows only one gap in 43.6–43.9 Mb in this study, and the presence of a rare density of SNPs (< 10) was observed, particularly in the intergenic region and between 42.1–44.8 Mb.

This approach is more efficient if tag SNPs based on the HapMap JPT r^2 ^structure are used to find the susceptibility variant [28]. We estimated the captured tag SNPs as proxies, meaning that the SNP showed a strong correlation with one or more SNPs at r^2 ^> 0.7, 0.8, or 0.9. The density of most tag SNPs was < 10 SNPs per 300-kb bin (Figure 1). Taken together, the coverage of common SNPs variation by our chosen SNP set was rather balanced in both gene-centric and intergenic regions, and the average number of common tag SNPs in this study was 30% of those in the HapMap JPT (r^2 ^> 0.8).

### Selection of a landmark SNP

**Figure 2:**
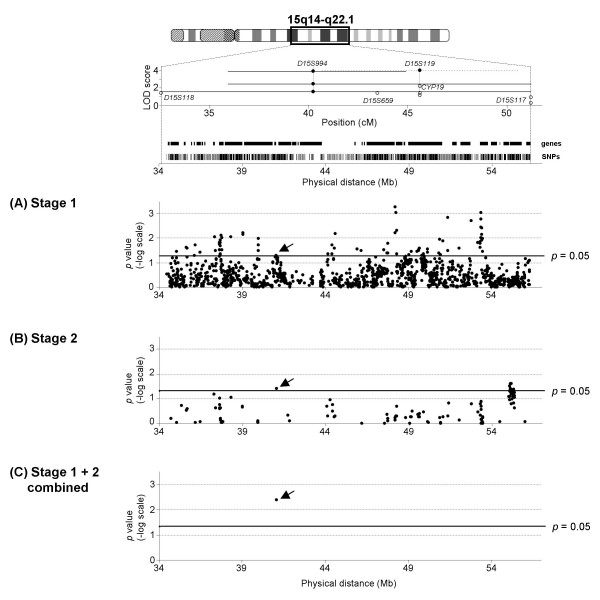
**Association tests on chromosome 15q14-22.1**. Allelic *p *values in association tests are shown. The top window shows reports of linkage with T2D or its related traits on chromosome 15q14-22.1 in the Japanese and other ethnic populations. The solid and open circles denote LOD scores and location in recombination distance. The width of the straight lines or a broken line denote a 1-LOD drop interval when available. The vertical bars show the positions of RefSeq genes (upper) and the 1,317 chosen SNPs (lower) with black vertical bars placed in gene-centric regions and gray vertical bars in intergenic regions. Allelic *p *values in Stage 1 (372 cases and 360 controls) for 1,317 SNPs (A), in Stage 2 (532 cases and 530 controls) for 112 SNPs (B), and in Stage 1 + Stage 2 combined samples (904 cases and 890 controls) for one replicated SNP (C) were plotted against physical positions. The data denote the -log *p*, and *p *= 0.05 is shown with a thick line. The landmark SNP2140 (rs2412747) is indicated with an arrow.

In Stage 1 association test, 112 SNPs out of 1,317 (8.5%) showed significant associations (χ^2 ^test, *p *< 0.05) in the allelic (Figure 2A) or genotypic test. In Stage 2, 11 SNPs out of 112 yielded significant associations (*p *< 0.05) in the allelic (Figure 2B) or genotypic test. However, only one SNP, namely SNP2140 (rs2412747), showed a replicated association for the allelic test (*p *< 0.05) in both stages (Figure 2C). These results suggest that our association test is underpowered. None of the other SNPs showed replicated association for genotypic frequency (Additional file 1).

When the raw data from Stage 1 and Stage 2 were combined, SNP2140 in intron 33 of the ubiquitin protein ligase E3 component n-recognin 1 (*UBR1*) gene exhibited the nominal significant association with T2D in both the allelic (*p = *0.0043, OR = 1.26, 95% CI = 1.07–1.48) (Figure 2C) and genotypic (*p = *0.0081) tests. However, a logistic regression analysis revealed that the association between SNP2140 and T2D was not statistically significant after adjustment for age, gender, and BMI. In addition, when the standard Bonferroni's correction for multiple testing was applied (corrected for 1,317 SNPs in the allelic and genotypic tests), the association with SNP2140 was no longer significant. Moreover, FDR for 112 SNPs in the Stage 2 association test was not significant at a threshold value of 0.1 [25].

The results of association for the top 20 Stage 1-associations using the Armitage trend test are shown in Table 3. Based on the combined *p *values, three SNPs (SNP3347: rs1386166, SNP3096: rs156787, and SNP2072: rs936216) showed a smaller *p *value than the landmark SNP2140 in the allelic test (Table 3). However, none of the three SNPs showed significant association in Stage 2, although sample size and power to detect susceptibility SNPs was larger than in Stage 1. We believe that the SNP with replicated significance in both Stage 1 and Stage 2 is regarded as the more likely SNP for T2D association. Unfortunately, none of 112 SNPs reached the statistical confidence level in our sample population.

**Table 3:** Top 20 SNPs and the landmark SNP2140 in Stage 1 allelic and genotypic association tests

						Stage 1					Stage 2					Stage 1 + Stage 2				
								
	SNP Marker	rs#	Gene symbol	Position	Allele 1/2	Allele 1 frequency case	Allele 1 frequency control	*p *value of allele frequency	*p *value of genotype frequency	*p *value of Armitage trend test^a)^	Allele 1 frequency case	Allele 1 frequency control	*p *value of allele frequency	*p *value of genotype frequency	*p *value of Armitage trend test^a)^	Allele 1 frequency case	Allele 1 frequency control	*p *value of allele frequency	*p *value of genotype frequency	*p *value of Armitage trend test^a)^
1	SNP2248	-	*ATP8B4*	48175632	G/T	0.23	0.31	**0.00053**	**0.0024**	**0.00052**	0.29	0.28	0.59	0.61	0.59	0.27	0.30	0.075	0.19	0.075
2	SNP2372	rs4261468	*RAB27A*	53342894	A/G	0.49	0.41	**0.00091**	**0.0034**	**0.00078**	0.47	0.48	0.67	0.80	0.66	0.48	0.45	0.076	0.20	0.072
3	SNP3295	rs12443171	intergenic	48225107	C/T	0.34	0.43	**0.00091**	**0.0030**	**0.00099**	0.38	0.40	0.49	0.66	0.49	0.37	0.41	**0.0079**	**0.018**	**0.0081**
4	SNP3347	rs1386166	intergenic	51376772	A/G	0.35	0.44	**0.0014**	**0.0013**	**0.0016**	0.37	0.40	0.15	0.23	0.16	0.36	0.42	**0.0017**	**0.0080**	**0.0021**
5	SNP1439	rs9972478	*RAB27A*	53347024	A/C	0.30	0.23	**0.0017**	**0.0068**	**0.0016**	0.27	0.29	0.28	0.34	0.27	0.28	0.26	0.25	0.37	0.25
6	SNP3391	rs2414351	intergenic	52746565	C/G	0.24	0.18	**0.0019**	**0.0066**	**0.0017**	0.19	0.21	0.16	0.39	0.17	0.21	0.20	0.34	0.64	0.35
7	SNP2373	rs2305424	*RAB27A*	53368640	C/T	0.47	0.55	**0.0037**	**0.011**	**0.0032**	0.48	0.48	0.97	0.86	0.96	0.48	0.51	0.061	0.11	0.058
8	SNP1441	rs2414409	*PIGB*	53419009	C/T	0.47	0.55	**0.0039**	**0.013**	**0.0034**	0.48	0.48	0.93	0.91	0.93	0.48	0.51	0.077	0.16	0.074
9	SNP1280	rs2278167	*SLC27A2*	48285140	G/T	0.61	0.54	**0.0050**	**0.014**	**0.0047**	0.59	0.56	0.20	0.095	0.19	0.60	0.55	**0.0054**	**0.013**	**0.0047**
10	SNP2039	rs2412424	*FSIP1*	37765425	C/T	0.65	0.59	**0.0075**	**0.022**	**0.0061**	0.62	0.62	0.91	0.62	0.91	0.64	0.61	0.072	0.16	0.067
11	SNP2098	rs4924524	*CHAC1*	39042673	C/T	0.76	0.70	**0.0060**	**0.022**	**0.0064**	0.73	0.76	0.21	0.082	0.21	0.75	0.73	0.42	0.15	0.42
12	SNP1440	rs7183960	*PIGB*	53409987	C/T	0.47	0.54	**0.0074**	**0.022**	**0.0067**	0.48	0.48	0.80	0.93	0.79	0.48	0.50	0.13	0.26	0.13
13	SNP2249	rs11638841	*ATP8B4*	48203899	C/G	0.73	0.66	**0.0062**	**0.026**	**0.0069**	0.67	0.68	0.72	0.89	0.72	0.70	0.67	0.15	0.33	0.15
14	SNP3176	rs996215	intergenic	44577114	A/G	0.23	0.30	**0.0067**	**0.027**	**0.0073**	0.28	0.27	0.51	0.74	0.50	0.26	0.28	0.22	0.46	0.22
15	SNP2097	rs4924523	*CHAC1*	39042662	A/T	0.24	0.30	**0.0072**	**0.025**	**0.0076**	0.27	0.24	0.22	0.085	0.22	0.26	0.27	0.43	0.15	0.43
16	SNP2072	rs936216	*PAK6*	38344560	A/C	0.75	0.81	**0.0092**	**0.0075**	**0.0084**	0.75	0.78	0.093	0.14	0.10	0.75	0.79	**0.0032**	**0.015**	**0.0038**
17	SNP1438	rs12910930	*RAB27A*	53332998	C/G	0.33	0.27	**0.0095**	**0.016**	**0.0084**	0.28	0.31	0.20	0.25	0.20	0.30	0.29	0.49	0.24	0.49
18	SNP2041	rs2254829	*FSIP1*	37779957	C/T	0.66	0.59	**0.0099**	**0.030**	**0.0085**	0.63	0.62	0.84	0.67	0.84	0.64	0.61	0.071	0.17	0.068
19	SNP1444	rs3743203	*CCPG1*	53436254	C/T	0.47	0.53	**0.010**	**0.037**	**0.010**	0.47	0.47	0.98	0.83	0.98	0.47	0.49	0.11	0.22	0.10
20	SNP3096	rs156787	*FLJ39531*	37335619	A/G	0.18	0.23	**0.0092**	**0.038**	**0.011**	0.18	0.21	0.065	0.065	0.062	0.18	0.22	**0.0020**	**0.0041**	**0.0022**

*	**SNP2140**	**rs2412747**	***UBR1***	41074095	C/T	0.81	0.77	**0.049**	0.11	**0.049**	0.79	0.75	**0.039**	0.072	**0.039**	0.80	0.76	**0.0043**	**0.0081**	**0.0045**

Significant results (*p *< 0.05) and (*) SNP2140 (rs2412747) in the *UBR1 *are shown in bold type. a)Armitage trend test was performed by using the DeFinetti program http://ijg.gsf.de/cgi-bin/hw/hwal.pl. The positions of top 20 SNPs with their *p *values in the increasing order in Armitage trend test are almost the same as those in allelic test, except for SNP 2039, 1440, and 3096 of which *p *values were underlined.

We next searched for novel SNPs or mutation in the *UBR1 *gene. Two novel non-synonymous SNPs (M1359I in exon 37 and R1712H in exon 47) were identified by re-sequencing the *UBR1 *gene in 48 samples. The SNPs were not common (MAF = 0.02 and 0.01) and subsequent typing of all of the samples revealed no significance for these two variants in the allelic test (*p *= 0.17 and 0.96 for combined samples). To confirm our data, it will be necessary to test association using a larger number of independent samples.

### Fine LD mapping and a haplotype-based association test

Haplotype-based association tests assessed as a multi-locus test can be more sensitive for the detection of association than assessment by a single-locus association test. To perform this test, we examined LD structure with the pairwise |D'| values of the region, including the *UBR1 *gene. We searched for additional common variants around putative LD block boundaries with available database. Using the definition of |D'| > 0.95, we identified a large LD block consisting of 38 SNPs including seven additional SNPs spanning 355 kb across the *UBR1 *gene. None of the seven SNPs showed a significant association in Stage 1, Stage 2, or in the combined samples.

For fine mapping of the 355 kb LD block, we selected seven haplotype-tagging SNPs (htSNPs) from 38 SNPs that captured > 95% of haplotype frequencies comprising the respective block in all samples, and estimated a haplotype-based association test. A protective haplotype and at-risk haplotype showed a nominal permutation *p *value (0.005 and 0.03 for combined samples) with T2D (Additional file 2). However, after Bonferroni's correction (corrected for 1,317 SNPs multiplied by two + eight haplotypes), *p *values were not significant for either haplotype.

## Discussion

We applied a region-wide association test as an alternative to GWAS. This approach has been effective in the identification of candidate susceptibility genes in the target regions for T2D [19,20] and rheumatoid arthritis [33,34] in the Japanese population. We hypothesized that there was a high probability of detecting disease susceptibility genes with the use of evenly-spaced common SNP markers in the target region defined by replicated linkage evidence. We could not find any strong variant with single-locus association test or haplotype analysis in 1,794 Japanese subjects, although one SNP, SNP2140 (rs2412747), in intron 33 of the *UBR1 *gene showed nominal significance (*p *= 0.0043 for combined samples).

*UBR1 *encodes an E3 ubiquitin ligase of the N-end rule pathway, a conserved proteolytic pathway of the ubiquitin system whose substrates include proteins with destabilizing N-terminal residues. Recent data suggest that in Johanson-Blizzard syndrome (OMIM 243800), the pancreas exhibits pancreatic exocrine insufficiency and does not express *UBR1 *[35]. *Ubr1 *knockout mice exhibit decreased body weight or adipose tissue and an exocrine pancreatic insufficiency [35,36]. In addition, *UBR1 *mRNA is elevated in the atrophic muscles of diabetic rats [37]. At present, however, the pathophysiological mechanism by which the *UBR1 *influences T2D is unknown.

**Figure 3:**
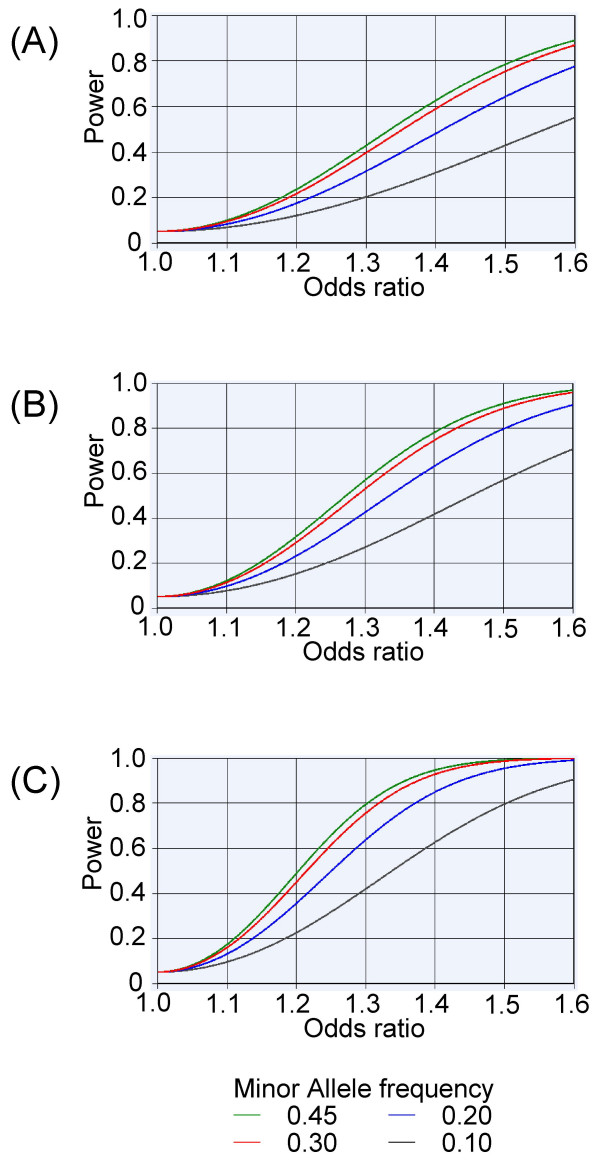
**Power calculation**. The power of this case-control association test to detect association against the risk allele frequency with a type 1 error rate of 0.05 in Stage 1 samples (A), in Stage 2 samples (B), and combined samples (C). 372 cases and 360 controls subjects were set in Stage 1, 532 cases and 530 controls subjects were set in Stage 2, and 904 and 890 controls subjects were set in Stage 1 + Stage 2 combined samples with variable power and a type 1 error rate of 0.05. A) – C) graphs were plotted with the PS program.

Genetic association tests aim to detect true positive associations between a trait and genetic polymorphisms while eliminating false positives. The results of this study have been evaluated within the framework of several criteria. Accordingly, the statistical power (with a MAF of 0.30) in Stage 1 or Stage 2 showed 22–60% or 29–76%, respectively, at detecting the OR of 1.2–1.4 at a significance level of 0.05 (Figure 3A, B). Although the power using the whole sample size was estimated as 45–93%, it was not sufficient to detect an association with T2D (Figure 3C). To achieve 60–80% power (with a MAF of 0.30 and OR of 1.2), 1,400–2,200 each for case and control samples (almost twice as many as those in each stage of this study) is needed. Recent GWAS analyzed more than one thousand cases and controls in Stage 1 and up to more than 10,000 samples in total [2-7], as recommended [38]. Replication studies with thousands of subjects are a priority to determine the consistency of our observations of the *UBR1 *gene, because it is predicted that true variant(s) have only a modest effect on T2D.

Our samples had the major weakness of an age difference between the case and control subjects (63.0 vs. 41.2). After logistic regression analysis, the association with SNP2140 did not remain statistically significant after adjustment for age and gender. Because aging is an important factor in diabetes development, it is necessary to confirm the result of our association test using age-matched control subjects. In selecting the target region for association assessment, the initial linkage studies might overestimate the relevance of chromosome 15q as a candidate region in the Japanese population. The linkage of chromosome 15q14-22.1 to T2D and its related traits have been replicated in the Japanese at a significant level (LOD = 3.91). However, this LOD score was observed in cases with young onset (< 45 years of age) T2D, which is not reflected in our cases (average age = 63.0).

Though SNPs markers were placed, on average, every 9,169 bp, which is comparable in density to a genome-wide association test using a 500 kb SNP set (~6 kb), it might be necessary to use denser SNPs with shorter intervals. Even though tag SNPs were not available at the onset of this study, tag SNPs with LD information and a high-density SNP map from the HapMap data would afford useful resources.

Based on these results, we propose that the discovery of a landmark SNP, SNP2140, in the *UBR1 *gene was statistically marginal and did not reach the confidence level of this study. Other SNPs did not exhibit a strong effect on the risk of T2D within the target region, despite suggestive replicated evidence for linkage in the Japanese population. In addition, a replication study is of prime importance for confirmation of these results.

## Conclusion

The two-stage association test on chromosome 15q14-22.1 failed to detect an association between T2D and SNPs in the target region. These observations could uncover new insights on the susceptibility variant(s) on chromosome 15q that might be used as a guide for further association tests, including replication and/or meta-analysis. Replication studies in other independent populations are a priority if the consistency of our observations is to be determined.

## Competing interests

The author(s) declare that they have no competing interests.

## Authors' contributions

YY carried out genotyping, sequencing, the association test, and LD analysis, and contributed to experimental design and manuscript writing. MM contributed to manuscript design and manuscript writing. DO, KN, and YF performed genotyping, performed the association test and LD analysis. TT, PK, and KK contributed to the development of statistical genetics concepts and maintained storage of the DNA samples. NN, TY, EI, HS, and NY supplied T2D DNA samples. HI and MI contributed to project design. All authors read and approved the final manuscript.

## Pre-publication history

The pre-publication history for this paper can be accessed here:



## Supplementary Material

Additional file 1 **Association tests in Stage 1, Stage 2, and Stage 1 + Stage 2**. A complete list of the 1,317 SNP markers used in this study, and the results of association tests in Stage 1, Stage 2, and Stage 1 + Stage 2. Click here for file

Additional file 2 **Haplotype-based association test with seven htSNPs by |D'|**. Inferred haplotype frequency and a haplotype-based association test on the landmark LD block across 355 kb. Click here for file
