# Supplementary material for: Lack of association of genetic variation in chromosome region 15q14-22.1 with type 2 diabetes in a Japanese population
Source: BMC Med Genet. 2008 Mar 27;9:22. doi: 10.1186/1471-2350-9-22 (PMC2324080; doi:10.1186/1471-2350-9-22)
Supplement: Additional file 2 — Haplotype-based association test with seven htSNPs by |D'|. Inferred haplotype frequency and a haplotype-based association test on the landmark LD block across 355 kb. [file 1471-2350-9-22-S2.doc]

|  | htSNPs^a)^ | | | | | | | Haplotype frequencies (%) | | | χ^2^ | *p* value | Permutation  *p* value^b)^ |
| --- | --- | --- | --- | --- | --- | --- | --- | --- | --- | --- | --- | --- | --- |
|  | rs10467975 | rs10518779 | rs6493068 | rs2412747 | rs2176870 | rs12594277 | rs11070392 | Case | Control | Overall |  |  |  |
|  | A/G | C/T | A/G | C/T | A/G | A/G | C/G | n = 904 | n = 890 | n = 1,794 |  |  |  |
| Hap-1 | G | C | A | C | A | G | G | 25.7 | 23.9 | 24.8 | 1.73 | 0.19 | 0.19 |
| Hap-2 | G | C | A | C | A | G | C | 17.3 | 18.4 | 17.8 | 0.76 | 0.38 | 0.40 |
| Hap-3 | G | C | A | C | A | A | C | 16.4 | 13.7 | 15.0 | 4.98 | **0.026** | **0.030** |
| Hap-4 | A | C | G | T | A | G | C | 13.7 | 17.0 | 15.3 | 7.54 | **0.006** | **0.005** |
| Hap-5 | G | T | G | C | G | G | C | 9.2 | 8.0 | 8.6 | 1.66 | 0.20 | 0.21 |
| Hap-6 | G | C | G | C | G | G | C | 6.9 | 7.5 | 7.2 | 0.51 | 0.48 | 0.49 |
| Hap-7 | G | C | G | T | A | G | C | 5.5 | 5.0 | 5.2 | 0.51 | 0.47 | 0.50 |
| Hap-8 | G | T | G | C | A | G | C | 1.9 | 2.1 | 2.1 | 0.18 | 0.67 | 0.73 |

**Additional file 2 Haplotype-based association test with seven haplotype-tagging SNPs (htSNPs) by |D'|.**

a) For fine LD block of 355 kb, eight haplotypes, which are sufficient to capture >95% of haplotype frequencies in all samples are shown with seven

haplotype-tagging SNPs (ht-SNPs) using 38 SNPs. This LD block included three genes: *UBR1*, tau tubulin kinase 2 (*TTBK2*), and transmembrane protein 62 (*TMEM62*).

b) The differences in the haplotype frequencies were analyzed by χ^2^-based *p* value, and permutation test (10,000 replicates) in all samples (904 cases, 890 controls) using SNPAlyze ver 5.1 Pro software.

Significant result (*p* <0.05) are shown in bold type.
